# Blastomycosis: A Review of Mycological and Clinical Aspects

**DOI:** 10.3390/jof9010117

**Published:** 2023-01-14

**Authors:** Kathleen A. Linder, Carol A. Kauffman, Marisa H. Miceli

**Affiliations:** 1 Division of Infectious Diseases, Department of Internal Medicine, University of Michigan Medical School, Ann Arbor, MI 48109, USA; 2 Veterans Affairs Ann Arbor Healthcare System, 2215 Fuller Road, Ann Arbor, MI 48105, USA

**Keywords:** blastomycosis, *Blastomyces dermatitidis*, *Blastomyces* antigen, itraconazole

## Abstract

Blastomycosis is caused by a thermally dimorphic fungus that thrives in moist acidic soil. *Blastomyces dermatitidis* is the species responsible for most infections in North America and is especially common in areas around the Great Lakes, the St. Lawrence Seaway, and in several south-central and southeastern United States. Other *Blastomyces* species have more recently been discovered to cause disease in distinct geographic regions around the world. Infection almost always occurs following inhalation of conidia produced in the mold phase. Acute pulmonary infection ranges from asymptomatic to typical community-acquired pneumonia; more chronic forms of pulmonary infection can present as mass-like lesions or cavitary pneumonia. Infrequently, pulmonary infection can progress to acute respiratory distress syndrome that is associated with a high mortality rate. After initial pulmonary infection, hematogenous dissemination of the yeast form of *Blastomyces* is common. Most often this is manifested by cutaneous lesions, but osteoarticular, genitourinary, and central nervous system (CNS) involvement also occurs. The diagnosis of blastomycosis can be made by growth of the mold phase of *Blastomyces* spp. in culture or by histopathological identification of the distinctive features of the yeast form in tissues. Detection of cell wall antigens of *Blastomyces* in urine or serum provides a rapid method for a probable diagnosis of blastomycosis, but cross-reactivity with other endemic mycoses commonly occurs. Treatment of severe pulmonary or disseminated blastomycosis and CNS blastomycosis initially is with a lipid formulation of amphotericin B. After improvement, therapy can be changed to an oral azole, almost always itraconazole. With mild to moderate pulmonary or disseminated blastomycosis, oral itraconazole treatment is recommended.

## 1. Introduction

Blastomycosis is an infection caused by thermally dimorphic fungi of the genus *Blastomyces*; these organisms exist as molds in the environment but as yeasts in tissues at body temperature. There are now known to be multiple species of *Blastomyces,* with each having a specific geographic distribution. The most common species is *B. dermatitidis*, for which clinical, diagnostic, and treatment aspects have been extensively described. The focus of this review will be on *B. dermatitidis*; the epidemiological and mycological characteristics of the other less common species will be mentioned briefly.

## 2. Epidemiology and Mycology

The ecological niche preferred by *B. dermatitidis* is moist, acidic soil near waterways, such as rivers and lakes [1,2]. The organism is considered endemic in the midwestern, southeastern, and south-central United States bordering the Mississippi and Ohio River basins, the St. Lawrence Seaway, and the Canadian provinces of Ontario, Manitoba, and Saskatchewan. It should be noted that blastomycosis is not a reportable infection, and thus the true prevalence and incidence are not known. 

Most infections are acquired sporadically from an environmental source, but outbreaks in which several to many people have been infected are well described in both rural and urban settings [3,4,5,6]. Activities associated with outbreaks usually involve disruption of soil by construction or excavation work or outdoor recreation activities, such as camping, fishing, or hunting [3,4,7,8]. One large outbreak stands apart from those typically described; this outbreak involved a cluster of cases in neighborhoods in central Wisconsin. Almost half of all cases in this report were among persons of Hmong ancestry, most of whom denied outdoor activities such as those noted above, raising questions about a possible genetic predisposition to infection [9,10]. 

In highly endemic areas, dogs, especially those used for hunting, are frequently infected and may develop severe, fatal blastomycosis [4,8,11,12]. Other mammals, such as cats, are infrequently infected, most likely because exposure is minimal in comparison with dogs which are more likely to have nose to ground. 

Recent advances in genetic analysis of the genus *Blastomyces* have clarified the nomenclature for agents that cause blastomycosis [13]. (Table 1) Initially, disease was thought to be caused only by *Blastomyces dermatitidis*. However, more sophisticated phylogenetic analysis of isolates of *Blastomyces* revealed a novel cryptic species, *Blastomyces gilchristii*, which is most commonly found in areas of northern Wisconsin, and western Ontario [14]. *B. dermatitidis* and *B. gilchristii* are genetically distinct species, but they are indistinguishable by any other means, and for the purpose of this review, the *B. dermatitidis/gilchristii* complex will hereafter be referred to as *B. dermatitidis*. 

Occasional cases of blastomycosis have been reported in Africa and the Middle East. Although it was initially postulated that these cases were also due to *B. dermatitidis*, recent genetic analysis suggests that these cases are more often caused by *Blastomyces percursus* or *Blastomyces emzantsi* [15]. *B. percursus* occurs throughout the Middle East and Africa, and cases of *B. emzantsi* have been reported only in South Africa to date; *B. dermatitidis* only rarely causes disease in this part of the world [16]. Manifestations of infection with *B. percursus* and *B. emzantsi* are clinically distinct from those of infection caused by *B. dermatitidis.*

In 1970, a case of blastomycosis was diagnosed based on clinical findings in Alberta, Canada, outside the typical range for *B. dermatitidis* [17]. The yeast phase of the organism, albeit unusual, was most consistent with blastomycosis. However, the organism could not be converted from the mycelial phase to the yeast phase and did not sporulate. This was originally thought to represent an atypical strain of *B. dermatitidis*, but later analysis of this isolate as well as several others from western Canada and the western United States led to identification of the species as *Blastomyces helicus* [18]. The tissue yeast phase of *B. helicus* is slightly smaller than *B. dermatitidis*, and instead of a single broad-based bud, multiple buds are attached to the mother cell. Unlike *B. dermatitidis* and *B. percursus*, *B. helicus* does not produce conidia, but instead forms characteristic coiled hyphae in the mold phase. 

Two additional species, *Blastomyces parvus* and *Blastomyces silverae*, have also been reclassified under this genus. *B. parvus* was previously identified as *Emmonsia parva* and causes adiaspiromycosis in rodents; sporadic human cases have been reported in heavily immunocompromised persons [19]. *B. silverae* has not been implicated in human disease [15]. 

## 3. Clinical Manifestations

### 3.1. Pulmonary Blastomycosis

As the lung is the primary route of entry for *Blastomyces* conidia, it is not surprising that symptomatic patients primarily manifest pulmonary symptoms [20]. However, data from large exposure cohort studies suggest that up to 50% of persons who are infected with *B. dermatitidis* are asymptomatic [3]. Symptoms begin between two weeks and several months after an exposure to conidia, but an obvious exposure event is not always identified. Reactivation of prior infection and reinfection have both been described but are uncommon [21]. 

The presentation of blastomycosis mimics other etiologies of community-acquired pneumonia, and the diagnosis may be delayed if a fungal etiology is not considered [22]. The symptoms include cough, fever, night sweats, and fatigue [23,24]. In acute pulmonary blastomycosis, radiographs and computed tomography (CT) usually show patchy infiltrates, but consolidation with air bronchograms may also be observed. With less acute infection, imaging can show mass-like lesions mimicking cancer or nodules of varying sizes; cavitary disease resembling reactivation tuberculosis and miliary disease patterns have also been described [25,26]. 

Overwhelming primary infection with *B. dermatitidis* uncommonly leads to diffuse pneumonitis and adult respiratory distress syndrome (ARDS) [27,28]. Patients with ARDS due to blastomycosis typically present with the sudden onset of fevers, cough, and dyspnea. Hypoxemia quickly ensues, and intubation and mechanical ventilation are required. Mortality rates have been reported to vary from 40% to 89% [28,29]. 

### 3.2. Extrapulmonary Manifestations

Dissemination of *Blastomyces* outside the lungs occurs in between 20% and 50% of cases. The most common site of dissemination is to the skin, followed by involvement of osteoarticular structures and the genitourinary system [20,23]. Spread to the central nervous system (CNS) is uncommon and can present either as isolated CNS disease or as part of widely disseminated infection. 

Skin lesions due to *Blastomyces* almost always occur secondary to hematogenous spread [30]. Skin lesions may appear after the pneumonia has cleared or concomitantly as part of widespread disseminated infection. In rare cases, infection has occurred secondary to direct cutaneous inoculation [31,32]. The cutaneous lesions are typically verrucous, with heaped-up borders and punctate areas of purulence in the center, and without significant surrounding erythema. Biopsy classically demonstrates microabscesses and pseudo-epitheliomatous hyperplasia. However, in some patients, cutaneous ulcerations, rather than granulomatous lesions, are noted. 

Osteoarticular disease is the second most common extrapulmonary manifestation of disease [20,33,34,35]. Blastomycosis usually manifests as disease in a single bone, of which the vertebrae and the long bones are most affected [33,35]. Lytic or sclerotic bone lesions can be confused with metastatic disease [34]. Joints can be infected by spread from a contiguous cutaneous or subcutaneous lesion. In autopsy series, *Blastomyces* is reported to frequently involve the genitourinary system, but symptomatic infection is less common and mostly manifested as prostatitis or epididymo-orchitis [23]. 

Blastomycosis uncommonly involves the CNS and may present either with signs and symptoms of a mass lesion or as a chronic meningitis [36]. CNS manifestations are more common in patients who have disseminated blastomycosis, and neurologic symptoms in someone with known blastomycosis should prompt further evaluation with targeted CNS imaging and a lumbar puncture. However, isolated CNS infection, either chronic meningitis or brain abscess, can occur without other signs of blastomycosis [36]. 

### 3.3. Blastomycosis Due to Less Common Species

Blastomycosis due to *B. percursus* seems to have a higher rate of extrapulmonary dissemination than that due to *B. dermatitidis*; cutaneous findings are very common, and isolated cutaneous disease is described in about 25% of patients [15,16]. Pulmonary disease has been noted in just over half of the reported cases, and osteoarticular disease, including fistulae to bones and joints, has also been reported. Cases of *B. emzantsi* are rare and mostly described in the profoundly immunocompromised. Case reports have described both pulmonary findings as well as the involvement of the subcutaneous tissues and bone [15,16]. 

*B. helicus* has been implicated in several cases of blastomycosis in both humans and animals from western Canada and the western United States [18]. Additionally, some cases that were previously thought to be caused by *B. dermatitidis* or *Emmonsia* spp are suspected to represent *B. helicus* infection based on retrospective review. Most reported human cases occur in those who are immunocompromised. Pulmonary findings appeared frequently, most commonly as diffuse nodular disease, and the organism was identified in the blood in 50% of cases. Mortality was 71% in one series [18]. 

## 4. Diagnosis

Diagnosis rests solidly on knowing the typical clinical picture seen with blastomycosis and integrating that with the knowledge that this is an organism acquired from certain areas in the environment that foster its growth. Thus, a careful history detailing both current and past residence, travel, military service, occupations, and avocations should be sought. Remote history regarding these points is also relevant because latent infection, although uncommon, can lead to reactivation infection. Most patients will have some history of living, working, or traveling to rural areas that are endemic for *B. dermatitidis*. Events such as harvesting dead trees, moving large quantities of soil, or exploring moist woodland areas near fresh water are often linked to blastomycosis. Dogs are quite susceptible to blastomycosis, and the development of pneumonia in a pet dog, especially one used for hunting, should trigger the possibility that pneumonia or skin lesions in the owner might be signs of blastomycosis [11,37]. 

### 4.1. Culture

Isolation of *B. dermatitidis* in culture provides proof that this is the organism causing infection [38]. Appropriate material for culture includes sputum or bronchoalveolar lavage fluid in patients with pneumonia; synovial fluid in those with articular infection; cerebrospinal fluid in patients with meningitis; and biopsy samples taken from skin, prostate, or other involved tissues [39,40]. Laboratory methods entail plating the sample on an appropriate medium, such as Sabouraud’s dextrose agar, and incubating at 25–27 °C. Growth may take up to 4 weeks to manifest as an off-white mold. The original method for defining a mold as *B. dermatitidis* involved converting the mold form to the yeast form on special agar at 35–37 °C. Most laboratories do not use this labor-intensive technique but prefer a commercially available DNA probe assay (AccuProbe; Hologic Inc., San Diego, CA, USA) that quickly and easily establishes the mold as *B. dermatitidis* [41]. Not surprisingly, both *B. gilchristii* and *B. helicus* test positive with this probe [18]. 

### 4.2. Histopathology and Cytology

**Figure 1:**
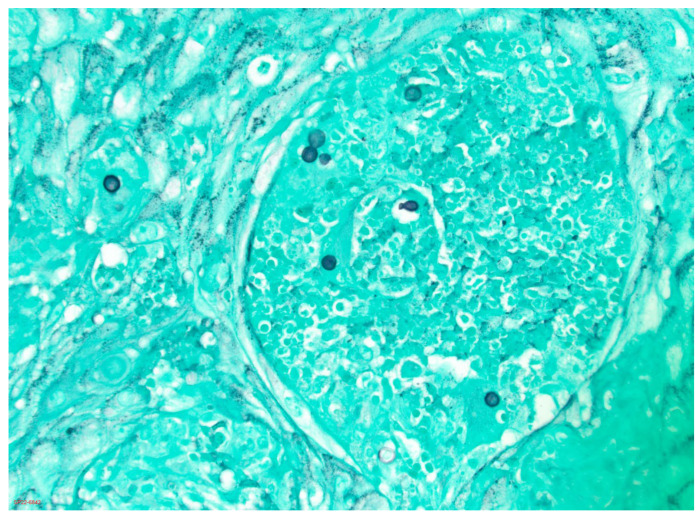
Grocott methenamine silver stain of biopsy of an exophytic lesion on the face of a middle-aged man. Thick-walled yeasts approximately 8–10 μm in diameter, one of which shows broad-based budding typical of *B. dermatitidis*, are seen (20× magnification).

**Figure 2:**
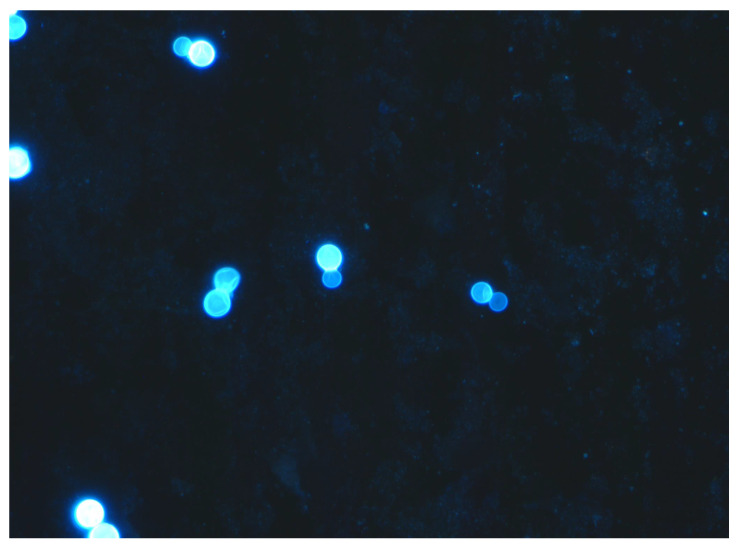
Calcofluor white stain of bronchoalveolar lavage fluid from a patient with severe pulmonary blastomycosis highlighting multiple thick-walled broad-based budding yeasts (40× magnification).

An equally efficacious method for establishing a diagnosis of blastomycosis involves identification of the typical yeast phase form of *B. dermatitidis* in tissues by histopathology or in fluids by cytologic methods. The advantage of this approach is that it provides a rapid diagnosis while waiting for culture results to be reported. This is especially important in patients who have a severe illness and need treatment quickly. The yeast form of *B. dermatitidis* is distinctive enough to prove the diagnosis of blastomycosis [38]. The organism is relatively large (generally 8–15 µm), has a thick wall, and has the distinction of having the single, budding daughter yeast-cell stay attached with a broad base to the mother yeast cell until the two cells are almost equal in size. Tissues should be stained with methenamine silver stain or periodic acid Schiff (PAS) stain (Figure 1). Body fluids can be viewed using calcofluor white stain (Figure 2); sputum is often stained with Papanicolaou stain, which readily shows the large yeast forms of *Blastomyces* [37,39]. Very rarely, the yeast forms of *B. dermatitidis* are smaller than expected and can be confused with other pathogenic yeasts. In this instance, use of a Congo Red stain, which stains *Blastomyces*, but not other yeasts, is helpful [39].

### 4.3. Antigen Detection

An enzyme immunoassay (EIA) (Mira Vista Diagnostics, Indianapolis, IN) has proved useful in the rapid diagnosis of blastomycosis. This assay detects galactomannan that exists in the cell wall of *B. dermatitidis*, and the assay is usually performed on urine or serum [42,43]. Performing this assay using BAL fluid can be helpful in the diagnosis of pulmonary blastomycosis [44,45]; additionally, the assay can be carried out using cerebrospinal fluid if a patient is suspected of having central nervous system blastomycosis [36,46]. Several studies have reported that sensitivity of the assay in urine has varied from 76% to 90% and in serum from 56% to 82% [42,43,47,48]. The test is most useful in patients who have more severe disease with a greater burden of infecting organisms, and it has been used effectively in dogs that often have disseminated infection [11,12]. It is important to note that there is a high degree of cross-reactivity between the *Histoplasma* and *Blastomyces* EIA tests [42,43], confirming the similarity between the galactomannans in the cell walls of these two genera. Thus, a patient with blastomycosis will often have a positive EIA for *Histoplasma*, as well as *Blastomyces*, and the reverse also occurs. Most times, the concentration of antigens is usually higher for the organism causing the infection. A few cases have been reported in which patients with blastomycosis have had not only a positive *Blastomyces* antigen in serum, but also a positive *Aspergillus* galactomannan EIA in serum (Platelia; Bio-Rad, Hercules, CA, USA) [49,50]; however, patients with aspergillosis have not been reported to have a positive *Blastomyces* EIA.

The utility of following *Blastomyces* antigen levels in urine to assess the response to therapy has been noted in a very few reported patients [51,52]. More experience has been reported in canines in which a decrease in urine *Blastomyces* antigen levels correlates with response to therapy [11,12,53]. 

### 4.4. Antibody Tests

Development of a sensitive and specific antibody assay to aid in the diagnosis of blastomycosis has been a difficult task that has stretched over several decades. The classic approach measuring antibodies by immunodiffusion and complement fixation has not proved useful for the diagnosis of blastomycosis [54,55]. Subsequent assays that measured the antibody response to the BAD-1 adhesion antigen on *B. dermatitidis* were an improvement [56]. The most sensitive assay to date, reportedly with a sensitivity of 88% in one report, also used the BAD-1 antigen, but with an EIA method (MiraVista Diagnostics, Indianapolis, IN) [57]. Use of this assay in dogs with blastomycosis showed sensitivity as high as 95% [58]. Whether this EIA will gain widespread use in the clinical approach to the diagnosis of blastomycosis in humans remains to be seen.

### 4.5. Nucleic Acid Tests

Polymerase chain reaction (PCR) methods have high specificity and are increasingly used for diagnosis of many infections. They have the potential to be useful in establishing a diagnosis of blastomycosis and in differentiating different species of *Blastomyces* [59]. Studies have been completed on both fresh and paraffin-treated tissues samples and also on soil samples [60,61,62]. Currently, PCRs are available for clinical use from only a few reference laboratories [63]. Nucleic acid tests will likely become standardized and more readily available in the future.

## 5. Treatment

All symptomatic persons with blastomycosis should receive antifungal treatment regardless of the severity of symptoms or site of infection [64]. If untreated, patients with blastomycosis are at high risk for progression or recurrence of this infection. The treatment of blastomycosis consists of either amphotericin B or an azole drug, and the approach to therapy will depend on the site and severity of infection, as well as the patient population (i.e., immunosuppressed host, pregnant people, and children). 

### 5.1. Mild to Moderate Pulmonary or Disseminated Blastomycosis without CNS Involvement

The first-line therapy for mild to moderate blastomycosis is oral itraconazole [64,65]. Patients should receive a loading dose of 200 mg three times daily for 3 days, followed by 200 mg twice daily. For patients with mild pneumonia, treatment should extend for 3–6 months, depending on their clinical and radiological response to therapy. For patients who have an extrapulmonary infection, therapy should continue for 6–12 months, and those with osteoarticular infection should receive a minimum of 12 months of therapy to avoid relapse [64]. 

Therapeutic drug monitoring of itraconazole is recommended due to the variable serum concentrations in patients receiving either capsule or liquid formulation itraconazole. A serum itraconazole concentration should be obtained after 2 weeks of therapy when steady state has been reached. It is important to note that most laboratories measure serum itraconazole concentrations using a high-performance liquid chromatography assay that is reported back as itraconazole concentration and hydroxy-itraconazole concentration. Because the metabolite hydroxy-itraconazole has antifungal activity, the total concentration of active drug is the sum of these two values. Serum concentrations of 1–2 µg/mL are recommended. When serum concentrations exceed 4 µg/mL, toxicity is more frequently seen [66].

Absorption of itraconazole capsules is enhanced by food and gastric acidity. The capsules should be taken with a meal; an acidic beverage, such as a cola drink, can increase absorption. In contrast, itraconazole solution has better bioavailability and does not require gastric acidity for absorption; thus, it should be taken on an empty stomach. For this reason, itraconazole solution is required for patients who must take H2-blockers or proton-pump inhibitors. If itraconazole is not tolerated or absorption is problematic, other azole agents can be used, as noted below.

### 5.2. Moderately Severe to Severe Pulmonary or Disseminated Blastomycosis without CNS Involvement

The initial treatment of moderately severe to severe blastomycosis without CNS involvement consists of a lipid formulation of amphotericin B, 5 mg/kg/d [64]. Treatment with amphotericin B should continue for a few weeks until clinical improvement is noted. This should be followed by itraconazole (loading dose of 200 mg three times daily for 3 days, then 200 mg twice daily) for at least 12 months. In order to reach steady state more quickly, it is prudent to start the loading doses of itraconazole before discontinuing amphotericin B. 

Patients who have blastomycosis-associated ARDS have a high mortality rate. Adjunctive therapy with intravenous methylprednisolone, 1 mg/kg/day for 7 to 10 days, is often used in these severely ill patients. Although no controlled clinical trials have been carried out, several reports suggest a clinical benefit related to the anti-inflammatory effect of corticosteroids [27,67,68]. Extracorporeal membrane oxygenation (ECMO) has also been used in the management of patients with blastomycosis-associated ARDS. Published data to support this practice come from small case series and case reports that have shown variable clinical response [69,70,71,72,73]. In one report, occlusion of the ECMO circuit during the administration of liposomal amphotericin B was noted and was associated with an unfavorable outcome [73]. Whether use of amphotericin B deoxycholate would obviate this problem is not known. Thus, the role of ECMO in the management of blastomycosis-associated ARDS needs further study. 

### 5.3. Central Nervous System Blastomycosis

CNS blastomycosis, whether isolated or one component of disseminated infection, and whether meningitis or a discrete focal lesion, should be treated initially with a lipid formulation of amphotericin B, 5 mg/kg daily, for 4–6 weeks. This is followed by treatment with an azole antifungal agent for a total of at least a year [64]. However, optimal duration of antifungal treatment is unknown and ultimately depends on the response to therapy and the patient’s immune status. In some patients, lifelong therapy with an azole may be required to prevent relapse [64]. Although initial recommendations from the IDSA Guidelines Panel for Management of Blastomycosis recommended either fluconazole or itraconazole, these recommendations have been supplanted by increasing experience with voriconazole for this manifestation of blastomycosis [36]. Voriconazole has excellent CNS penetration and in vitro activity against *B. dermatitidis* and has been shown to be effective for the treatment of CNS blastomycosis in case series and individual case reports [74,75,76]. 

### 5.4. Alternative Antifungal Agents

A recently approved, highly bioavailable formulation of itraconazole (SUBA-itraconazole) enhances intestinal absorption, thus allowing lower dosing. Preliminary data have shown that SUBA-itraconazole at a dose of 130 mg twice daily achieved higher serum concentrations and area under the curve (AUC) without increased toxicity when compared with conventional itraconazole at a dose of 200 mg twice daily in patients with endemic mycoses [77]. 

Fluconazole is moderately effective for non-life-threatening blastomycosis. The experience treating patients with blastomycosis with fluconazole is limited to two small studies that showed treatment failures with fluconazole at a dose of 200–400 mg daily, and improved success rates using higher doses of 400–800 mg daily (65% vs. 87%, respectively) [78,79]. However, given its excellent penetration into the CNS, fluconazole can be used as stepdown treatment of CNS disease in patients who cannot tolerate voriconazole [46,80]. 

Voriconazole, as noted above, has become the preferred azole for the step-down treatment of CNS blastomycosis. This agent has also been reported to be effective for patients who have pulmonary or disseminated blastomycosis [81]. Therapeutic drug monitoring is essential when voriconazole is used to ensure that appropriate serum concentrations are achieved to ensure efficacy and to avoid several dose-related adverse effects, notably CNS and hepatic toxicity, seen when serum concentrations rise above 5.5 µg/mL [82]. 

Posaconazole has assumed a larger role in the treatment of blastomycosis, but there are no clinical trials comparing this agent with other azoles [83,84]. Posaconazole and itraconazole share similar structures, and posaconazole has excellent in vitro activity against *B. dermatitidis*. Absorption is much less problematic than with itraconazole or voriconazole, and toxicity appears to be less than that noted with the other agents. However, posaconazole should not be used when CNS involvement is suspected because of poor penetration into the CNS. As with itraconazole and voriconazole, therapeutic drug monitoring should be performed when posaconazole is prescribed to ensure serum concentrations are greater than 1 µg/mL [85,86]. 

A recent open-label, non-randomized clinical trial to define the usefulness of isavuconazole for treatment of invasive fungal diseases due to dimorphic fungi included only three patients with blastomycosis. Of these three, one person had complete resolution of infection, but the other two were treated only briefly and efficacy could not be evaluated [87]. Despite limited data to support the use of isavuconazole for the treatment of patients with blastomycosis, its favorable safety profile makes isavuconazole an attractive alternative when the use of other azoles is precluded [88]. 

Echinocandins, including the new agent rezafungin, are not active against *B. dermatitidis* and should not be used for the treatment of patients with blastomycosis. Olorofim is a novel oral antifungal that has demonstrated in vitro activity against dimorphic fungi, including *B. dermatitidis*. Clinical trials are currently ongoing, and preliminary results are promising [89]. 

### 5.5. Treatment of Blastomycosis in Special Populations

Severe pulmonary and disseminated infection, including CNS involvement and ARDS, is more likely to occur in immunocompromised patients than in immune competent persons. Therefore, solid organ transplant recipients and other immunocompromised patients with blastomycosis should be treated initially with a lipid formulation of amphotericin B [64]. Studies have shown that initial azole therapy in solid organ transplant recipients increases the risk of relapse and treatment failure [90,91,92]. After clinical response is noted, patients without CNS involvement can be transitioned to oral itraconazole, for at least 12 months of antifungal therapy. Length of therapy will depend on the extent of immune compromise. Long-term suppressive antifungal therapy with an azole in immunocompromised patients with blastomycosis is recommended. This recommendation is aimed to reduce relapse and recurrence in this patient population, but the level of evidence is low [64]. 

Pregnant people with any clinical form of blastomycosis should be treated with a lipid formulation of amphotericin B (3–5 mg/kg IV once a day). Azoles are teratogenic and should be avoided during pregnancy, especially in the first trimester [64]. 

Children tolerate several amphotericin B formulations better than adults. Therefore, children with moderately severe to severe forms of blastomycosis should be treated with either amphotericin B deoxycholate, 0.7–1.0 mg/kg daily, or a lipid formulation of amphotericin B, 5 mg/kg daily [64]. Children with mild to moderate disease should be treated with oral itraconazole; the dose given is usually 10 mg/kg twice daily.

## Figures and Tables

**Table 1:** Current species in the genus *Blastomyces*.

Species	Geographic Range	Notes
*B. dermatitidis*	North America, including midwestern, southern, southeastern United States, the St. Lawrence Seaway, and midwestern Canadian provinces	By far the most common etiology of blastomycosis
*B. gilchristii*	Primarily in certain areas of Wisconsin and Ontario	Only distinguishable from *B. dermatitidis* by phylogenetic analysis
*B. helicus*	Western Canada and western United States	Rarely causes disease, mostly in immunocompromised hosts
*B. percursus*	Middle East, Africa	More bone and cutaneous manifestations than classic blastomycosis
*B. emzantsi*	South Africa	Geographically restricted, but presents similarly to *B. percursus*
*B. parvus*	North and South America, Eastern Europe, Australia	Causative agent of adiaspiromycosis in rodents; rare infection in humans
*B. silverae*	Western Canada	Not implicated in human disease
